# The Impact of Lockdown During the COVID-19 Outbreak on Dietary Habits in Various Population Groups: A Scoping Review

**DOI:** 10.3389/fnut.2021.626432

**Published:** 2021-03-04

**Authors:** Grace Bennett, Elysia Young, Isabel Butler, Shelly Coe

**Affiliations:** ^1^Centre for Nutrition and Health, Department of Sport, Health Sciences and Social Work, Faculty of Health & Life Sciences, Oxford Brookes University, Oxford, United Kingdom; ^2^Centre for Movement Occupational and Rehabilitation Sciences, Department of Sport, Health Sciences and Social Work, Faculty of Health & Life Sciences, Oxford Brookes University, Oxford, United Kingdom

**Keywords:** diet, LockDown, COVID-19, lifestyle, fruit and vegetables

## Abstract

**Background:** Since the beginning of the COVID-19 pandemic, access to fresh food has been restricted, and people are spending more time inside and have limited their physical activity. However, more time at home may have resulted in some positive habits including an increase in cooking. The aim of this review was to assess dietary changes during the first lockdown. Themes and patterns were considered and associations with other lifestyle factors were assessed.

**Methods:** Between June and July 2020, the PubMed, Google Scholar, and Science Direct databases were searched, and results were screened for eligibility based on title, abstract, and full text. The inclusion criteria of this search included: papers published (or in pre-print) in the year 2020; studies that investigated the impact of COVID-19 lockdown on diet; papers published in English. Exclusion criteria were as follows: papers examining dietary changes in those following a structured diet based on diagnosed conditions or dietetic advice; literature, systematic, or narrative studies reviewing previous research. Researchers agreed on the study characteristics for extraction from final papers.

**Results:** Four thousand three hundred and twenty-two studies were originally considered with 23 final full-text papers included. Four themes were identified: dietary patterns, dietary habits (favorable), dietary habits (unfavorable), and other (includes physical activity levels, weight gain). A total of 10 studies reported an increase in the number of snacks consumed, while six studies found that participants increased their meal number and frequency during quarantine. Eleven studies reported favorable changes in dietary habits with an increase in fresh produce and home cooking and reductions in comfort food and alcohol consumption. However, nine studies found a reduction in fresh produce, with a further six reporting an increase in comfort foods including sweets, fried food, snack foods, and processed foods. Two studies reported an increase in alcohol consumption. In eight studies participants reported weight gain with seven studies reporting a reduction in physical exercise.

**Conclusion:** The effect of COVID-19 lockdown both negatively and positively impacted dietary practices throughout Europe and globally, and negative diet habits were associated with other poor lifestyle outcomes including weight gain, mental health issues, and limited physical activity. Both in the short term and if sustained in the long term, these changes may have significant impacts on the health of the population.

## Introduction

In March 2020, the World Health Organization (WHO) declared the coronavirus outbreak as a global pandemic (1). Currently, little is known of the transmission, mechanism, and treatment of COVID-19. It is believed that COVID-19 is spread via person-person contact, through respiratory droplets and because of this social distancing, hand washing, and wearing face coverings is crucial to reduce the spread of the virus (2).

As of July 2020, there have been over 570,000 global deaths related to COVID-19 (3). With no vaccine to prevent infection and no guaranteed treatment once infected, many countries have restricted public access to supermarkets, shops, and recreation facilities. The seriousness of restrictions varies among countries and is dependent on the incidence rates of infection. Despite lockdown measures taken by governments worldwide, over 13 million cases have been reported in over 200 countries (3).

Although such restrictions are to limit interpersonal contact and therefore transmission, many experts have voiced their concern for the long-lasting affects that lockdown may pose on individuals' mental and physical health (4). Lockdown has restricted the number of hours permitted for outdoor physical activity, people's access to fresh food, and previous research has shown that increased stress, which is likely during a global pandemic, can have a severe impact on an individual's lifestyle habits (5). Stress and anxiety have been shown to result in an increased intake of alcohol and sugary foods, and energy imbalance is also likely as energy expenditure during lockdown is reduced (5). However, positive lifestyle habits may also have emerged from the pandemic, including more time for cooking and reduced fast-food consumption.

Although there has been increasing and significant interest on the impact of the pandemic on various health outcomes, no review has addressed the association between COVID-19 and diet patterns globally. The aim of this review is to assess the literature on the impact of lockdown on dietary changes in various population groups. The objectives will be to assess diet patterns and themes in order to understand the dietary and associated lifestyle changes during the initial lockdown. It will be concluded how such changes may affect health and wellbeing in the short-term during the initial COVID-19 lockdown period and this will then inform research into the longer term effects of the pandemic on diet changes and health outcomes.

## Methods

This review followed the preferred reporting items for systematic reviews and meta-analyses extension for scoping reviews (PRISMA-ScR) and was in accordance with the JBI Manual for Evidence Synthesis: updated methodological guidance for the conduct of scoping reviews (6).

### Literature Search

A systematic literature search of the PubMed, Google Scholar, and Science Direct databases was conducted for studies published in 2020. Initially, the following search terms were used in the three databases to obtain a general understanding of the current research on the topic area: (COVID-19) AND (nutrition OR diet) AND (lockdown); (Food Habits) AND (Lockdown); (Dietary change) AND (COVID-19) AND (Lockdown). Following this search, alternative phrasing was noted among relevant studies and guidance on the search strategy was received. The search terms were then finalized and the following search terms were used in the systematic search: (Covid-19 OR Coronavirus) AND (Diet* OR Food OR Nutrition OR Eat*) AND (Lockdown OR Confinement OR Containment OR Quarantine OR Isolation). This search was undertaken between June 2020 and July 2020. No restrictors and filters were used in the database search so as to not inadvertently exclude any papers of interest.

### Selection Process

Results were screened for eligibility based on title, abstract, and finally full text. Two researchers independently screened the articles for eligibility (GB and EY) alongside the inclusion criteria. The inclusion criteria of this literature search were as follows:

1. Limit to papers published in the year 2020 (including pre-prints);
2. Studies that investigated the association of COVID-19 lockdown and diet;
3. Only research papers in English.

For this review, the exclusion criteria were as follows:

1. Papers examining dietary changes in those following a structured diet (e.g., diabetics/coeliacs) based on diagnosed conditions or dietetic advice;
2. Literature, systematic, or narrative studies reviewing previous research.

The search was kept broad in order to identify any relevant studies that would fit within the aims of the review, and as lockdown occurred during 2020, only papers within this year were searched. No authors were further contacted.

### Data Charting and Synthesis

Researchers GB and EY discussed search terms and databases prior to the initial search. All searches were documented in a shared excel spreadsheet accessible to all four researchers. After discussion among all four researchers, GB and EY performed a second and final search with the altered search terms in the same databases. Once eligible studies were identified based on the inclusion/ exclusion criteria, they were extracted and screened. An initial extraction template was developed and study characteristics were exported. The following characteristics from each study were extracted: first author, year, title, journal, type of study, participant number, age, location, findings, and conclusion. After consideration, researchers revised the extraction template to only focus on what they believed to be the important and relevant data from each study in regards to the study aims. A final extraction template of study characteristics for final full-text studies was agreed upon amongst all four reviewers. Results are shown in Table 1 and are composed of: study type/ design, assessment methods, location, gender, age, dietary change, and significant findings/findings that showed a change. Themes were agreed upon by all researchers and data from each paper for the relevant themes were gathered and grouped together for analysis.

**Table 1:** Overview of papers included in this review.

**N(ref)^1^**	**First author**	**Study type**	**Assessment method**	**Location**	**Female *N* (%)**	**Age (mean) in years**	**Dietary change**	**Findings that report a change and/or show significance**
(7)	Allabadi et al.	Cross-sectional study	Demographic, diet, and lifestyle survey (telephone)	Asia (Palestine)	300 (50%)	10–19 (14.1)	Comfort food, fruit, and vegetable increase	Food intake increased during lockdown; weight gain seen (*p* < 0.001). Increased food intake higher in females than males (*p* = 0.013).
(8)	ALMughamis et al.	Cross-sectional study	Demographic, diet, and lifestyle survey (WhatsApp)	Asia (India)	380 (72.8%)	(41.8)	Snacking, food intake increase	Change in dietary habits. Increased snacking, particularly after dinner. Weight gain seen in lockdown (*p* < 0.001). Increase in sedentary levels.
(9)	Ammar et al.	Cross-sectional study	Mental and physical health and lifestyle behavior survey (online)	Global (40% Africa, 36% Asia, 21% Europe)	563 (53.8%)	18+	Meal number, comfort food, and snacking increase	Increase in number of meals consumed (*p* < 0.001). Binge eating habits and snacking increased during confinement (*p* < 0.001). Consumption of unhealthy food increased (*p* < 0.001). Binge alcohol drinking decreased (*p* < 0.001).
(10)	Bhutani et al.	Cross-sectional study	Demographic, diet, and lifestyle survey (online)	Americas (U.S.^4^)	1,007 (56.6%)	18–75	Snacking, fruit, and vegetable increase	Increased intake of fruit and vegetables (*p* > 0.05). Increase in processed food intake and snacking (*p* > 0.001).
(11)	Bracale et al.	Panel study	Analysis of consumer trends	Europe (Italy)			Decrease in fresh food intake	Increase in long-life products: pasta, flour, eggs, homemade bread and pizza, and red or processed meat. Decrease in fresh produce.
(12)	Deschasau-Tanguy et al.	Cohort study	Demographic survey and 24-h recall (online)	Europe (France)	19,483 (52.3%)	15+ (52.1)	Snacking, comfort food increase, fresh food decrease	Change in dietary habits. Reported weight gain, particularly among young women. Increase in home cooking, snacking, comfort food, and alcohol intake. Reduction in fresh fruit, vegetable, meat, and fish.
(13)	Di Renzo et al.	Cross-sectional study	Demographic, diet, and lifestyle survey (online)	Europe (Italy)	2,486 (71%)	12–86	No change in meal number	Increase in homemade foods. Decrease in fresh fish, baked goods, and alcohol intake (*p* = 0.002).
(14)	Gallo et al.	Cohort study	Self-administered 24-h recall	Oceania (Australia)	295 (57.9%)	19–27	Snacking and food intake increase	Snacking and energy intake increase in females (*p* < 0.05). Increase in number of meals consumed at home (*p* < 0.0001). Reduction in walking (*p* < 0.05).
(15)	Husain et al.	Cross-sectional study	Demographic, diet, and lifestyle survey (online)	Asia (India)	285 (68.7%)	18–73 (38.5)	Snacking increase	Change in dietary habits, increase in number of meals (*p* = 0.000). Increase in snacking, particularly at night (*p* = 0.000). Increase in freshly made foods/home cooking. Decrease in red meat and fast food consumption (*p* = 0.000).
(16)	Matsungo and Chopera	Cross-sectional study	Demographic, diet, and lifestyle survey (online)	Africa (Zimbabwe)	318 (63%)	18+	Decrease in fruit and vegetables	Decrease in fruit, vegetables, nuts, seeds, cereals, breads, and tubers intake (*p* < 0.006). Increase in perceived body size seen in lockdown (*p* < 0.001).
(17)	Mehta	Cross-sectional study	Demographic, diet, and lifestyle survey	Asia (India)	25 (50%)	20–50 (37)	Snacking, meal numbers increase, fruit, and vegetable decrease	Change in dietary habits. Intake during meal times and snacking increased. Fruit and vegetable intake decreased during confinement.
(18)	Mitchell et al.	Retrospective cohort study	Dietary and lifestyle record (mobile app)	Americas (U.S.)	318,224 (83.4%)	18+ (47.8)	Decrease in fruit and vegetables	Decrease in fruit and vegetable intake. Increased in red and processed meat intake, particularly among men.
(19)	Parnham et al.	Cross-sectional study	COVID-19 related questionnaire	Europe (UK^5^)		8+	Reduction in school meals	Half of children did not receive free school meals during lockdown (*p* < 0.01).
(20)	Pellegrini et al.	Retrospective observational study	Demographic, diet, and lifestyle survey (telephone)	Europe (Italy)		18–75 (47.9)	Snacking increase	Increase in BMI and self-reported weight gain (*p* < 0.001). Lower educational levels, higher anxiety/depression levels, and poor dietary habits associated with weight gain (*p* < 0.001). Increased consumption of snacks, sweets, and cereals (*p* < 0.001).
(21)	Phillipou et al.	Cross-sectional study	Demographic, diet, and mood survey (online)	Oceania (Australia)	4,231 (80%)	18+	Food intake	Higher levels of binge eating (~35%) (*p* < 0.003), higher levels of restrictive eating (~28%) (*p* < 0.001).
(22)	Pietrobelli et al.	Cross-sectional study	Lifestyle questionnaire (in-person and telephone)	Europe (Italy)	19 (46%)	6–18 (14)	Meal number, comfort food increase	Increase in number of meals consumed (*p* < 0.001). Increase in fruit consumption (*p* = 0.055). Intake of crisps sweets and sugary drinks increased (*p* = 0.005 – <0.001).
(23)	Rodriguez-Perez et al.	Cross-sectional study	Demographic and dietary intake survey (online)	Europe (Spain)	5,305 (70.6%)	18+	No increase in meal intake	No increase in eating habits. Decreased alcohol intake and physical activity levels. Difficulty finding fresh fish and vegetables. Males had significantly different intake levels of fruit, vegetables, and olive oil (*p* < 0.006).
(24)	Romeo-Arroyo et al.	Cross-sectional study	Diet and lifestyle survey (online)	Europe (Spain)	301 (50.1%)	18–68 (42.6)	Comfort food increase	Increase intake of sweets. Decrease in fresh fish intake (*p* < 0.05).
(25)	Ruiz-Roso et al.	Cross-sectional study	Demographic, diet, and lifestyle survey (online)	Global (EU^2^ and South America)	495 (61.1%)	<14 to >17 (16)	Comfort food, fruit, and vegetable increase	Increase in fruit and vegetable (*p* < 0.0001) and legumes (*p* < 0.05) intake, higher compliance with recommendations. Fast food intake decreased (*p* < 0.0001). Sweet (*p* < 0.0001) and fried food (*p* < 0.001) intake increased.
(26)	Scarmozzino et al.	Cross-sectional study	Demographic and dietary intake survey (online)	Europe (Italy)		Anonymous	Comfort food, food intake increase	Increased eating during confinement. Increase in comfort food intake: chocolate, ice cream, and desserts. Increased snacking. Decreased alcohol intake.
(27)	Sidor et al.	Cross-sectional study	Demographic, diet, and lifestyle survey (online)	Europe (Poland)	1,042 (95%)	18+ (27.7)	Snacking and food intake increase	Increased eating and snacking during confinement in those with a higher BMI^3^ (*p* < 0.01). Highest BMIs had lowest fruit and vegetable intake, highest dairy, red meat, and fast food intakes (*p* < 0.05).
(28)	Zachery et al.	Correlational study	Demographic, lifestyle, and social survey (online)	Americas (U.S.)	96 (55.5%)	(26)	Snacking increase	Increased snacking after dinner (*p* < 0.001 and increased eating in response to stress or boredom (*p* = 0.041) in those who gained weight during lockdown.
(29)	Zhao et al.	Cross-sectional study	Demographic, dietary, and household food diversity survey	Asia (China)	1,273 (65.7%)	18+	No increase in meal intake	Increased intake of nutritional supplements. Younger age group had a lower diet diversity score.

1 N(ref), reference number;

2 EU, European Union;

3 BMI, body mass index;

4 U.S., United States;

5 *UK, United Kingdom; N, number*.

## Results

**Figure 1:**
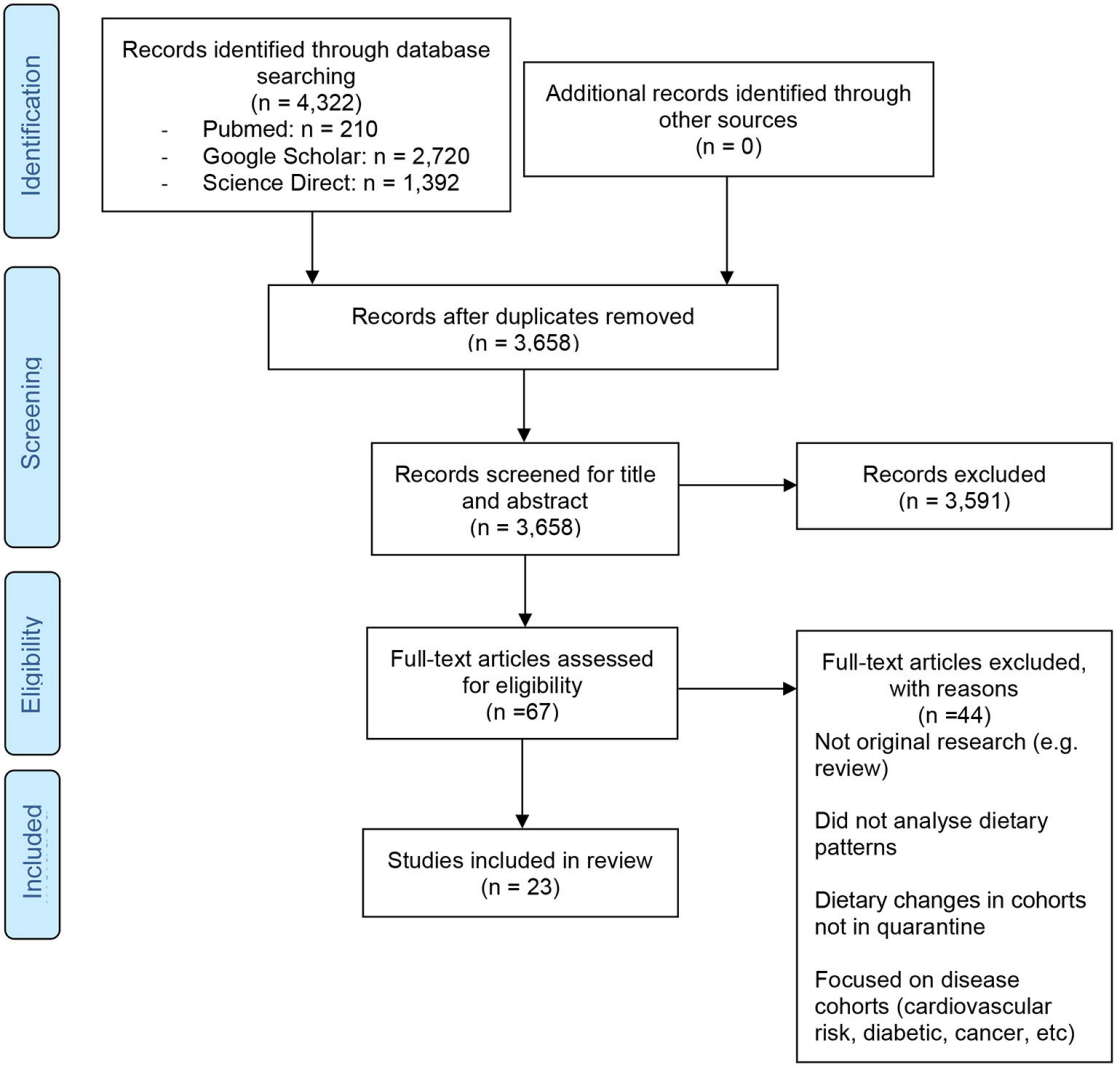
PRISMA 2009 flow diagram displaying the selection process of the 23 final papers.

Out of a total of 4,322 papers initially identified, 23 full papers were deemed eligible for the current review (Figure 1). Most studies included in this review are based on self-reported data among adult populations in the developed world. Seventeen studies were cross-sectional in nature (7–10, 13, 15–17, 19, 21–27, 29), and three cohort (12, 14, 18), one observational (20), one correlational (28), and one panel study (11) were included. Locations included global (9, 25), the US (10, 18, 28), Asia including Palestine (7), India (8, 15, 17), and China (29), Europe including Italy (11, 13, 20, 22, 26), France (12), Spain (23, 24), Poland (27), and the UK (19), Australia (14, 21), and Zimbabwe (16). The age range and total number of participants were not presented as these demographics were not stated in all the studies included in the review. A full overview of papers is included in Table 1, in which only results that showed a change were included. After reviewing all eligible papers, four research themes were derived from each paper for analysis and discussion purposes (Table 2). These included: dietary patterns (meal patterns and snacking), dietary habits (favorable), dietary habits (unfavorable), and other (includes physical activity levels, weight gain). The following discussion is separated into four parts, to support the research themes.

**Table 2:** Twenty-three papers in the review divided by theme and sub theme.

**Theme for discussion**	**Sub-sections**	**Paper reference numbers**
*Dietary patterns*		(7–9, 12–15, 17, 20–24, 26–28)
	*Snacking*	(8, 9, 12, 14, 15, 20, 24, 26–28)
	*Meal number*	(7, 12, 13, 15, 17, 21–23, 26, 27)
*Dietary habits: favorable*		(7, 9–13, 15, 22, 23, 25, 26)
	*Increase in fresh produce*	(7, 10, 22, 23, 25)
	*Increase in home cooking*	(11–13)
	*Reduction in comfort foods*	(15, 25)
	*Reduction in alcohol consumption*	(9, 23, 26)
*Dietary habits: unfavorable*		(7, 9–13, 16–18, 20, 22–26, 29)
	*Reduction in fresh produce*	(7, 10–13, 16–18, 23, 24)
	*Increase in comfort foods*	(9, 12, 20, 22, 25, 26)
	*Increase in alcohol consumption*	(12, 29)
*Other*		(7, 8, 12–14, 16, 19–21, 23, 26–29)
	*Reduction in physical activity*	(7, 8, 12, 14, 16, 21, 23)
	*Increase in supplementation*	(29)
	*Weight gain*	(7, 8, 12, 13, 20, 26–28)
	*School meals*	(19)

## Discussion of Results

### Dietary Patterns

#### Snacking

A significant change in snacking frequency and behaviors was reported by a total of nine studies that were reviewed (8, 9, 12, 14, 15, 20, 26–28, 30). ALMughanis et al. (8) reported an increase in snacking at night in 44% of participants. This trend was supported by three other studies that found that increased snacking behaviors were typically seen at night or after the last meal of the day (9, 15, 28). Late night food intake is viewed by many as an unfavorable dietary pattern as research has shown an association between late-night eating and the development of metabolic syndrome (31). Snacks mostly consisted of energy-dense and non-nutrient-rich foods. Scarmozzino et al. (26) reported that in Italy over 40% of participants increased consumption of “comfort foods” such as chocolate, ice cream, and desserts during lockdown. Romeo-Arroyo et al. (24) reported a 50% increase in sweets consumed. Three papers (14, 20, 28) found that snacking was used as a mechanism to help cope with increased anxiety levels during self-quarantine. Following this finding, promotion of other avenues of stress relief, including physical activity and meditation, may be influential when advising population groups how to maintain sustainable and healthy lifestyle habits.

#### Meal Number

Six studies reported that participants saw an increase in their meal number and frequency during lockdown (7, 15, 17, 22, 26, 27, 30). In Mumbai, India, Mehta et al. (17) completed a 15-question survey with 50 participants and found that over half of participants increased their meal consumption, with a large portion choosing foods that had a low preparation time and meals based around ready-to-eat foods and animal produce. In a study by Deschasaux-Tanguy et al. (12), 37,252 adults from France completed a lockdown-specific questionnaire on nutritional behaviors before and after lockdown, between April and May 2020. This research found that although food intake increased, diet diversity and fresh produce, such as fruit and vegetables, intake reduced. Three of the studies investigated did not report an increase in food consumption (13, 21, 23). Phillipou et al. (21) found that almost 30% of participants had restricted their eating habits during confinement, primarily due to weight gain concerns. Husain et al. (15) found that in Kuwait, almost 42% of their cohort skipped breakfast during quarantine, which increased slightly from 39% before lockdown.

### Change in Dietary Habits: Favorable

#### Increase in Fresh Produce

A total of 11 papers reported favorable changes in dietary habits during the coronavirus pandemic lockdown (7, 9–13, 15, 22, 23, 25, 26, 32). Pietrobelli et al. (22) identified an increase in fruit consumption in 41 children and adolescents in Italy (*p* = 0.055). An increase in fruit intake is supported by four other studies (7, 10, 23, 25). Ruiz-Roso et al. (25) found an increase in both fruit and vegetable intake in 820 adolescents during confinement. An explanation for this could be the increase in home cooking and the promotion from the WHO of the importance of fruit and vegetables during confinement. In 7,154 Spanish adults, an increase in fruit and vegetable intake during confinement was observed in a higher proportion for males than females compared to pre-confinement (23). In contrast, Bhutani et al. (10) reported a higher proportion of females in the United States (US) increasing fruit and vegetable consumption in confinement compared to US males. The difference in results could be due to Spanish female participants having a higher adherence to the Mediterranean diet (MedDiet), which is high in fruit and vegetables, prior to lockdown, and therefore any increase would be more noticeable in a US female cohort.

#### Increase in Home Cooking

Based on consumption trends, there was an increase in the amount of home cooking with a significant increase in foods such as homemade pizza and bread (11). This is supported by Deschasaux-Tanguy et al. (12) and Di Renzo et al. (13) who both reported an increase in home cooking. The increase in home cooking could be attributable to various reasons including reduced availability of eating out and/or fast food options, increase in family time, safety concerns, and finally it could be used as a method to overcome boredom.

#### Reduction in Comfort Foods

A decrease in total fast food consumption during lockdown has been reported in participants in Kuwait, Spain, Italy, Brazil, Colombia, and Chile (15, 25). In a cohort of 415 participants living in Kuwait, 82.2% did not consume any fast food during lockdown and this dietary habit reportedly continued post lockdown with 13.7% consuming fast food 1–2 times per week compared to 49.4% pre-lockdown. Consideration needs to be taken for the fact that many fast food outlets would have been closed during lockdown which would have significantly affected fast food intake.

#### Reduction in Alcohol Consumption

Ammar et al. (9) found via a short dietary behavior questionnaire that the largest dietary habit change during lockdown was a reduction in binge drinking. It is thought that the younger participants in the study would have limited access to alcohol and less social interaction with friends which may have impacted the results. Social incentives have been found to be more of an influence over coping motives for increased alcohol consumption in the younger population and therefore this may have caused a significant reduction on drinking in this group during the pandemic (33). A 36.8% reduction in alcohol consumption in Italy was reported, however this is thought to have been overexaggerated due to an underreporting of alcohol intake which is often associated with self-reporting questionnaires (26). However, Rodríguez-Pérez et al. (23) identified a lower intake of alcohol during lockdown in Spain and reported that these dietary changes continued with a higher adherence to the MedDiet post lockdown.

### Change in Dietary Habits: Unfavorable

#### Reduction in Fresh Produce

Nine papers assessed the reduction in fresh produce (7, 10–13, 16–18, 24). Overall the papers showed a reduction in purchasing and consumption of fresh produce. Bracle et al. (11) assessed consumer purchase trends from February to March 2020 in an Italian population and found a dramatic reduction in fresh goods including fruit and vegetables. Similarly, Deschasaux-Tanguy et al. (12) found that 27.4% of participants reported buying less fresh products due to poorer access to usual food stores and/or products. Rodriguez-Perez et al. (23) reported just over a quarter (27%) had issues with buying certain foods which included meat (23.83%) and vegetables (13.86%).

Research by Matsungo and Chopera (16), using a cross-sectional online survey for 507 people living in Zimbabwe, found that 94.8% of people reported an increase in food price with decreased availability (64%) and a decrease in quality (43.9%), during a 4-week lockdown in Zimbabwe, showing the massive effect COVID-19 has had on the food chain supply system. Therefore, price or financial status may also play a role in reduced purchase as Mehta (17) found that 27% of participants reduced fruit and vegetable intake and although unavailability was a key reason, 32% reported that the increase in price of these consumables influenced their lack of purchase and/or consumption. Allabadi et al. (7) performed telephone interviews with 600 adolescents in Palestine. In total, 36.7% increased fried food intake and 46.5% increased sweets and sugar-added food intake, and this was associated with weight gain and worsening financial status during lockdown.

As mentioned above, stress and anxiety may have significant influence over food habits during lockdown as Mitchell et al. (18) found through a self-reported food intake questionnaire in 381,564 participants pre and during COVID-19 lockdown. Fruit (men −4.2%, women −5%), salad (men −5.9%, women −6.5%), vegetables (men −4.7%, women −6.3%), and lean protein (men −3.6%, women −3.9%) intake decreased. These results were compared to other studies in which stress during “natural disasters” has been shown to correlate with reduced fresh produce consumption (30). Matsungo and Chopera (16) found food prices increased and availability decreased. Over the 4-week lockdown period, 57.8% said they reduced vitamin A rich fruit and vegetable intake, 48.5% decreased other vegetable intake, and 64.9% decreased other fruit intake, with a reduction in fresh produce associated with higher anxiety.

#### Increase in Comfort Foods

Pietrobelli et al. (22) found that the consumption of unhealthy foods including potato chips, red meat, and sugary drinks significantly increased 3 weeks into lockdown in a sample of 41 young people in Italy who were classified as obese (*p* < 0.005–0.001). Ruiz Roso et al. (25) used an online questionnaire in a multi-country cohort of adolescents and found sweet food and fried food consumption increased during COVID-19 confinement compared to before COVID-19. There were significant country differences and those who watched TV during mealtimes had higher sweet food and fried food consumption. In another study, an increased consumption of sweets and chocolate (22%) and biscuits and cakes (20%) was found, which was associated with other poor diet habit changes (12). Therefore, multiple factors may have increased the intake of unhealthy or comfort foods during COVID-19 lockdown, including already being overweight or obese, more sedentary time at home, and an overall change in dietary habits/patterns.

In an international sample of 1,047 participants, unhealthy food consumption had increased during confinement, which was due to changes in mood including lack of motivation and anxiety and/or boredom (9). Scarmozzino et al. (26) found that, similar to Ammar et al. (9), the increase in negative eating behaviors was thought to be due to increased anxiety in this population during lockdown (42.7%). Pellegrini et al. (20) used a questionnaire in 150 obese participants in Northern Italy and found that unhealthy food consumption, specifically sweets intake increased by 50%. This increase was associated with weight gain during lockdown which may have been related to increased anxiety.

#### Increase in Alcohol Consumption

Two studies looked at increased alcohol consumption during lockdown (12, 29). Zhao et al. (29) used a cross-sectional questionnaire-based survey in 1,938 Chinese participants to assess what lifestyle changes people used to “cope” with COVID-19 and found that 10.6% of participants increased their alcohol consumption intentionally. In another study, when considering food groups, alcoholic beverage consumption increased in 15% of the participants and decreased in 12%. The increase in alcohol intake was associated with other unhealthy changes in diet patterns, with higher anxiety and depression scores and with working from home (12).

### Other

#### Reduction in Physical Activity

During a 4-week lockdown in Zimbabwe, Matsungo et al. (16) reported that 62.5% of people surveyed reduced their physical activity. This may be explained by an increase in screen time in 89.1% of respondents. Similarly, Rodriguez-Perez et al. (23) showed that while people's dietary pattern did not dramatically deviate from the MedDiet during lockdown, over 59.6% decreased their levels of physical activity. Three papers (7, 8, 12) showed that at least 50% of people surveyed had decreased levels of exercise in Kuwait, Palestine, and France due to confinement. An Australian study by Phillipou et al. (21) reported that in the general population group the majority of people (43.4%) decreased their exercise levels, which may be related to working from home and restrictions on sporting activities and gym closures during the peak of the pandemic. Gallo et al. (14) carried out a survey on US students comparing data from 2018/2019 to data collected during the 2020 lockdown. Although vigorous exercise levels remained the same, students reported a decrease in walking during 2020, which may be related to no longer commuting to campus or walking between classes. Examining a subsection of biomedical students, 80% were classed as “sufficiently active,” which dropped to 62% for men and 55% for women in 2020, showing the negative effect lockdown has on physical activity levels among young adults.

#### Increase in Supplementation

Zhao et al. (29) showed that 37.7% of people surveyed intentionally consumed certain foods, supplements, or Chinese herbs as they thought it would help against COVID-19. These people had a higher Household Dietary Diversity Score which the paper concluded could mean they were more “in tune” with their health. People mainly consumed vitamin C (18.2%), however despite government advice, some people consumed alcohol (10.6%) or vinegar (16%) under the false assumption that consumption would kill or help fight the virus.

#### Weight Gain

Three Italian surveys noted increased weight gain among participants related to the consumption of certain foods (13, 20, 26). In Northern Italy, Pellegrini et al. (20) reported a significant increase of 1.51 kg on average among participants. Those of lower education levels, disinterest in the “healthiness” of foods, and who self-reported anxiety and depression were more likely to report weight gain. Di Renzo et al. (13) reported 48.6% of people saw an increase in weight, which may be due to an increased appetite during lockdown. Scarmozzino et al. (26) showed the lowest reported weight gain (19.5%). In the US, Zachary et al. (28) reported a lower number of people (22%) reporting weight gain but a larger increase in weight, ~5–10 pounds (2.2–4.5 kg). Weight gain was linked to increased eating due to stress (52%), boredom (73%), seeing or smelling food (65%), snacking after dinner (65%), being around friends and family (65%), and cravings (52%). Four other papers reported an increase in weight due to changes in the type of food consumed (7, 8, 12, 27). ALMughanis et al. (8) reported in Kuwait that there was a significant change in weight (1.13 kg), which is unsurprising as 74.1% claimed they changed their dietary habits. Deschasaux-Tanguy et al. (12) showed that participants (35%) gained weight (1.8 kg) due to reduced physical activity and an increase in calories by over 10%. Energy intake vs. energy expenditure will have shifted during the initial lockdown and the resulting weight gain following these patterns is therefore not surprising. However, this new “normal” of confinement at home may necessitate the need to find alternative ways of managing weight, diet, and exercise, especially if continued (32).

#### School Meals

Parnham et al. (19) surveyed children eligible for free school meals (FSM) in the UK and found that children on FSM were more likely to receive vouchers during the 1st month of lockdown in the UK than those through the universal scheme. Secondary school children were more likely to receive the voucher than children from junior schools, but this could be due to them being classified by means testing for FSM not through the universal scheme. There was also a correlation between receiving FSM and the household visiting a food bank.

### Dietary Changes by Country

Country specific changes in diet and lifestyle habits were also assessed in the current review to determine if any patterns were apparent. In Australia, Gallo et al. (14) and Phillipou et al. (21) both reported a reduction in physical activity during the initial lockdown. In the US, Bhutani et al. (10) and Mitchell et al. (18) assessed the reduction in the consumption of fresh produce, with only Mitchell et al. showing this trend. ALMughanis et al. (8) and Husain et al. (15) showed an increase in snacking behavior in the Indian population. In Italy, Bracale et al. (11) and Di Renzo et al. (13) showed evidence for an increase in home cooking yet a reduction of fresh produce, while Pellegrini et al. (20), Pietrobelli et al. (22), and Scarmozzino et al. (26) reported an increase in comfort foods. Di Renzo et al. (13), Pietrobelli et al. (22), and Scarmozzino et al. (26) asked participants if there were changes in the number of meals consumed with only Pietrobelli et al. and Scarmozzino et al. reporting an increase in the numbers of meals, while Di Renzo et al. found no change. Also, Di Renzo et al. (13), Pellegrini et al. (20), and Scarmozzino et al. (26) showed that participants reported weight gain. In Spain, Rodriguez-Perez et al. (23) and Romeo-Arroyo et al. (24) both reported a reduction in consuming fresh produce. Finally, Ammar et al. (9) and Ruiz-Roso et al. (25), which both covered multiple continents, both showed an increase in consumption of comfort foods. Therefore, although it is difficult to conclude anything definite, there seems to be agreement between studies within the same country and this could potentially be due to the different lockdown timelines and policies implemented in these locations. For example, over half of the countries in this review entered either a full or partial lockdown by the end of March 2020, with the government restricting movement and social interaction, which may have had a sudden and significant impact on the frequency of accessing supermarkets to buy fresh produce, the lack of food-associated social occasions, and limiting exercise.

### Strengths and Limitations

Previous research has highlighted the negative effects that lockdown has on health, however, this study is focused on the thematic analysis of dietary change and provides a unique insight into how the COVID-19 pandemic has impacted dietary behavior globally. The evidence included in this review is from a short time period in 2020 and therefore the studies are based on acute changes to diet and associated lifestyle factors. Even though this review is a starting point for considering long term effects of COVID-19 on health, so far this can only be predicted from the short-term studies. Only studies with full-text publications and published in English were considered for this review, which may lead to selection bias. As with most nutritional research, dietary intake was assessed through self-reported data, where misreporting, most commonly underreporting, is possible. Studies in this review were predominately cross-sectional in design and therefore the risk of bias and the quality of each study was difficult to assess due to the nature of the review and the studies included. It was not possible to assess quality compared to longer term cohort/ cause-effect research. Only findings that showed a change were reported in the results, as anything that did not significantly change did not differ compared to prior to lockdown. This review had limitations in its classification of a scoping review due to the short-term nature of the studies included and the limited literature in which to discuss the findings, however it was felt that this review type was the most appropriate for the current topic. Pre-print research papers were included in this review (8, 10, 12, 17, 18) and therefore despite having a DOI, these papers had not undergone peer-review prior to being included in this review. The search terms were not fact checked by a librarian and therefore the search strategy was constructed based on the expertise of the four authors of this review, who between them have an extensive background in the nutrition research field and in writing systematic and scoping reviews.

## Conclusion

Although some studies noted a decrease in food consumption and healthier diet practices during lockdown, many studies found either an increase in snacking and meal numbers, or an increase in unfavorable food choices and dietary habits. Therefore, COVID-19 lockdown resulted in both favorable and unfavorable changes in eating practices, and this may have both short- and long-term consequences on health. Although over half of the studies included European populations, there is evidence to suggest that action is required globally to encourage people to re-adopt healthy lifestyle habits during and post lockdown.

The positive diet practices that resulted from lockdown included an increase in the consumption of fresh produce, mostly fruit and vegetables, and an increase in home cooking during lockdown. However, poor food habits were seen in the majority of studies including increased snacking and meal frequency, reduced fresh produce, an increase in comfort foods, and alcohol intake. Reasons for negative changes in food behavior predominately included limited availability and increased price, and there were associations with poor food choices and mental health conditions including depression and anxiety, and sedentary time and weight gain. Those with a higher BMI were more likely to develop unhealthy dietary habits during quarantine (27). The increase in negative food choice such as alcohol intake was associated with other unhealthy changes in diet patterns, with higher anxiety and depression scores and with working from home (12). Conversely, those who showed a healthier overall diet routine throughout lockdown were shown to exhibit other healthy behaviors such as a reduced alcohol intake and increased supplement use. Reduced physical activity and weight gain were the main concerns found in some studies, and alongside poorer eating habits these factors may overall cause significant public health worry.

If negative dietary patterns are sustained post lockdown, they may contribute to health issues among population groups in the future, as being overweight and obese leaves individuals more susceptible to chronic health conditions and disease. Indeed, sedentary behavior including increased screen time and television viewing is associated with unhealthy eating patterns, and therefore this is a pattern that is likely seen during confinement (34). It is also a possibility that future lockdowns may occur, and therefore changes in eating habits may continue. Although positive changes in dietary practices were seen during the first lockdown, the impact of COVID-19 on lifestyle behaviors including diet was detrimental across populations, and therefore future research should aim to help the recovery and maintenance of healthy lifestyle habits to prevent the long-term health effects of the pandemic.

## Author Contributions

All authors listed have made a substantial, direct and intellectual contribution to the work, and approved it for publication.

## Conflict of Interest

The authors declare that the research was conducted in the absence of any commercial or financial relationships that could be construed as a potential conflict of interest.

## Abbreviations

COVID-19: Coronavirus disease
FSM: free school meals
MedDiet: Mediterranean diet
PRISMA-ScR: preferred reporting items for systematic reviews and meta-analyses extension for scoping reviews
WHO: World Health Organization.
