# Supplementary material for: The Impact of Lockdown During the COVID-19 Outbreak on Dietary Habits in Various Population Groups: A Scoping Review
Source: Front Nutr. 2021 Mar 4;8:626432. doi: 10.3389/fnut.2021.626432 (PMC7969646; doi:10.3389/fnut.2021.626432)
Supplement: Supplementary file 1 [file Table_1.DOCX]

Appendix 1

*Full search strategy for PubMed database*

Search terms (1)

(COVID 19) AND (nutrition OR diet) AND (lockdown)

(Food Habits) AND (Lockdown)

(Dietary change) AND (COVID-19) AND (Lockdown)

No filters were applied.

Search terms (2)

(Covid-19 OR Coronavirus) AND (Diet* OR Food OR Nutrition OR Eat*) AND (Lockdown OR Confinement OR Containment OR Quarantine OR Isolation)

No filters were applied.
